# Extraction of Novel Effective Nanocomposite Photocatalyst from Corn Stalk for Water Photo Splitting under Visible Light Radiation

**DOI:** 10.3390/polym15010185

**Published:** 2022-12-30

**Authors:** Nasser A. M. Barakat, Aya Gamil, Ibrahim Ashour, Khalil Abdelrazek Khalil

**Affiliations:** 1 Chemical Engineering Department, Faculty of Engineering, Minia University, El-Minia 61519, Egypt; 2 Department of Mechanical & Nuclear Engineering, College of Engineering, University of Sharjah, Sharjah 27272, United Arab Emirates

**Keywords:** corn stover, water photo-splitting, nano catalysts, hydrogen, renewable energy

## Abstract

Novel (Ca, Mg)CO_3_&SiO_2_ NPs-decorated multilayer graphene sheets could be successfully prepared from corn stalk pith using a simple alkaline hydrothermal treatment process followed by calcination in an inert atmosphere. The produced nanocomposite was characterized by SEM, EDX, TEM, FTIR, and XRD analytical techniques, which confirm the formation of multilayer graphene sheets decorated by inorganic nanoparticles. The nanocomposite shows efficient activity as a photocatalyst for water-splitting reactions under visible light. The influence of preparation parameter variations, including the alkaline solution concentration, hydrothermal temperature, reaction time, and calcination temperature, on the hydrogen evolution rate was investigated by preparing many samples at different conditions. The experimental work indicated that treatment of the corn stalk pith hydrothermally by 1.0 M KOH solution at 170 °C for 3 h and calcinating the obtained solid at 600 °C results in the maximum hydrogen production rate. A value of 43.35 mmol H_2_/gcat.min has been obtained associated with the energy-to-hydrogen conversion efficiency of 9%. Overall, this study opens a new avenue for extracting valuable nanocatalysts from biomass wastes to be exploited in hot applications such as hydrogen generation from water photo-splitting under visible light radiation.

## 1. Introduction

Biomasses are being produced in massive amounts everywhere. For environmental issues, instead of conventional disposal strategies, this large amount of waste is exploited to produce biogases (e.g., methane) [1,2,3], liquid fuels (e.g., ethanol) [4,5,6], or new chemicals (e.g., polyamides) [7,8,9]. Mineral constituents, which form ashes during biochemical conversion or thermal treatment processes, exist in almost all kinds of biomasses in different compositions and contents. For instance, pines’ pulp has deficient mineral compounds (0.5%), miscanthus and corn stover have about 8–10%, and rice hulls have ash contents as high as 21% [10,11,12]. These physiologically bound materials are bound in the cell walls and incorporated into the vascular structure.

These metal-containing compounds create problems during the processing of biomasses. During biochemical conversion, increased mineral content directly correlates to a reduction in the carbohydrate content of the feedstock, reducing sugars’ yield [13]. Furthermore, high mineral content has an unfavorable effect on the product output in many thermochemical conversion processes, such as liquefaction, pyrolysis, and fast pyrolysis. In boilers and the heat transfer surfaces of biomass gasifiers, high alkali and silicon contents contribute to fouling and slagging, which strongly decreases the overall thermal efficiency [11,12]. Moreover, in pyrolysis, alkali and earth alkali metals drastically decrease levoglucosan production [14,15]. Accordingly, pretreatment is essential before processing the biomasses in many biochemical and thermal conversion processes. Therefore, several pretreatment strategies have been developed in order to remove the mineral compounds from the biomasses either by extraction or neutralization. For example, hot deionized water [16], strong acid (0.1 wt% HNO_3_) [17], and 2 wt% phosphoric acid [18] were utilized.

Paradoxically, these physiologically bound minerals, which result from intrinsic biomass properties such as plant type, maturity, and anatomical fractions, contain important metals which can be utilized valuably in other applications. Consequently, the main target of this study is extracting an efficient photocatalyst from abundant agricultural waste to be invoked in green hydrogen production from water splitting under visible light radiation.

Corn is one of the most widely planted crops. For instance, the corn produced worldwide was over 1.2 × 10^9^ metric tons in the 2021/22 marketing year. Consequently, a vast amount of corn stover is produced annually. This biomass included cobs, sheaths, leaves, stalks, and husks. Stalks are the principal constituent representing about 60% of the dry mass and at least 50% of the total stover carbohydrate. Stalks consist of nodes and internodes; the latter represents two-thirds of the entire stalk’s dry and carbohydrate mass [19]. Although several processes have been developed to reutilize corn stover, such as livestock feedstuff, energy source, pulp, and fertilizer, a large amount of this biomass is burnt in open fields. Environmental regulations prohibit this process because it causes severe problems such as air pollution, fire disasters, surface water pollution, and potential safety concerns [20,21]. The metal content in the corn stover has been estimated and is summarized in Table 1 [10]. As shown in the table, other elements exist in relatively high amounts besides silicon, the dominant element. These elements can form valuable semiconductor composites. Moreover, if the treatment process could be adjusted to formulate the carbon in the form graphene, this can add more advantages to the produced composite.

Water photo-splitting under visible light radiation is considered to be the most desirable green hydrogen production technique from an economic and environmental points of view. The photocatalyst is the backbone of this process [22]. Although titanium-based compounds were widely utilized as photocatalysts for water splitting [23], some other elements also showed good catalytic function toward this hydrogen-producing reaction, such as Ca, Al, Mg, Fe …, etc. [24,25,26,27,28,29]. Moreover, supporting the functional materials on proper carbonaceous supports strongly enhanced the photocatalytic performance [30,31]. Therefore, corn stover might be an excellent precursor to produce an effective photocatalyst for the water photo-splitting process. It contains essential metals, and its carbon content can be formulated into a nanostructural morphology if proper treatment is performed.

Corn stover is a lignocellulosic material mainly composed of cellulose (~35% *w*/*w*), hemicellulose (~20% *w*/*w*), and lignin (~12% *w*/*w*) [32]. Lignin and other aromatic compounds present in the plant cell walls are the primary determinants of biodegradation. The pretreatment step is mainly achieved by removing these materials from the cellulosic materials. Alkali treatment results in the saponification of ester linkages between the cell wall components, which is an effective pretreatment process [33].

This study successfully produced effective inorganic NPs/graphene composite from the corn stalk by a simple alkali hydrothermal treatment process followed by sintering in an inert atmosphere. The produced composite showed distinct performance as a photocatalyst for splitting water under visible light radiation.

## 2. Materials and Methods

### 2.1. Catalyst Preparation

The corn stalk was collected from the local farms in the countryside in Minya governorate, Egypt. First, the corn stalk was cut into small pieces (approximately 1 cm in length), washed with distilled water, and dried at 60 °C for 12 h before use. The corn stalk shell and pith were utilized to check the biomass source. Within a subsequent hydrothermal treatment, 4 g of the dry corn stalk was mixed with 150 mL of homogeneous potassium hydroxide (KOH, Al-Safa company, El Minya, Egypt) aqueous solution. The slurry was then put in a 280 mL Teflon-lined stainless-steel autoclave and subjected to thermal treatment. After the hydrothermal treatment, the slurry was filtered and washed many times with distilled water to remove the residual KOH and the water-soluble impurities. The dried cake was sintered at elevated temperatures. The influence of several parameters on photocatalytic activity has been studied, including hydrothermal time, hydrothermal temperature, alkaline solution concentration, and calcination temperature.

### 2.2. Characterization

The surface morphology was examined using a scanning electron microscope (Hitachi S-7400,Tokyo, Japan) outfitted with an EDS analysis instrument. The phase and crystallinity were identified using a Rigaku X-ray diffractometer C (XRD, Rigaku, Japan) with Cu Kα (λ = 1.540 Å) radiation throughout a Bragg angle range of 10° to 80°. Transmission electron microscope (TEM, JEOL JEM-2010, Akishima-Shi, Japan) pictures with standard and high resolution were reported. The Batch mode of Shimadzu’s GC-2025 capillary gas chromatograph was used for gas detection. A special syringe was used to inject the gas into the instrument. The Raman spectrum has been plotted using DXR3xi Raman Imaging Microscope, Thermo Fisher Scientific, USA. X-ray photoelectron spectroscopy analysis (XPS, AXIS-NOVA, Kratos Analytical Ltd., Manchester, UK) was utilized to check the surface composition. The investigation was conducted at a base pressure of 6.5 × 10^−9^ Torr, resolution (Pass Energy) of 20 eV, and scan step of 0.05 eV/step. Fourier-transform infrared (FT-IR) spectroscopy (PerkinElmer, Waltham, MA, USA) was used to analyze the bonding arrangements of the samples.

### 2.3. Hydrogen Production Experiment

A 500 mL Pyrex spherical glass was used to perform hydrogen production experiments. A precisely measured amount (100 mg) of the prepared catalyst was added to 100 mL of distilled water–methanol solution (1:1 by volume). The plug was sealed and connected with a rubber tube in a conical flask, then the measuring cylinder was filled with water and turned upside-down in a glass beaker. The mixture was exposed to a halogen lamp (2000 watt) as a visible light simulator. Continuous stirring, to maintain constant homogeneity of the suspension, was carried out using a magnetic stirrer with a magnetic bar. The used data were obtained from the average results of three consequent experiments.

## 3. Results

### 3.1. Catalyst Characterization

**Figure 1:**
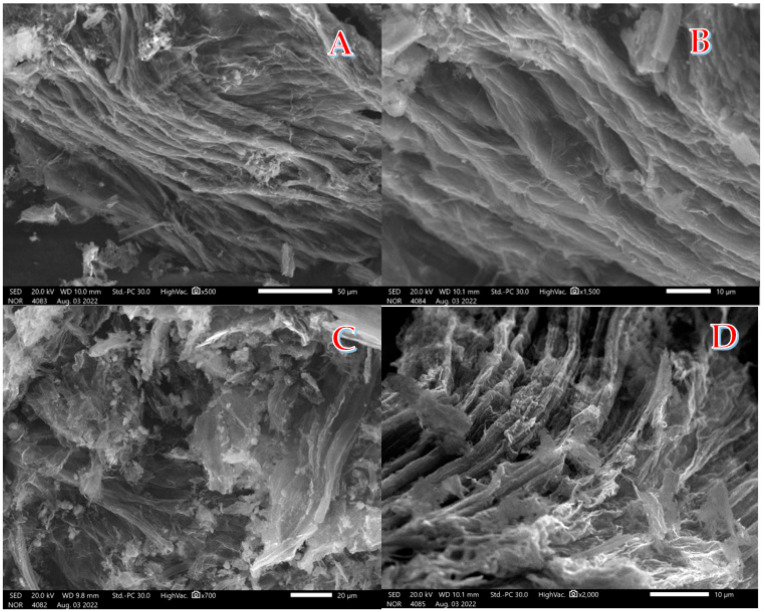
Two magnification SEM images of the powder produced from hydrothermal treatment using 3.0 M KOH at 150 °C for 6 h and calcined at 800 °C; (**A**,**B**), and at 1.0 M KOH at 170 °C for 3 h and calcined at 600 °C; (**C**,**D**).

Due to its complex structure, lignin cannot be degraded during the biological or biochemical conversion of the biomasses to produce either gaseous or liquid simple hydrocarbons. Moreover, in the case of utilizing biomasses as precursors to produce nano carbons, its graphitization results in the formation of coarse graphite particles [34]. In this study, the hypothesis mentioned above was confirmed by the graphitization of a raw stalk pith; the obtained powder had large-size graphite particles. At the same time, an alkali hydrothermal treatment of the stalk pith with subsequent calcination in an inert atmosphere led to obtaining a very light carbonaceous material. Figure 1A,B depict SEM images of the powder produced by hydrothermal treatment of stalk pith at 150 °C for 6 h in the presence of 3.0 M KOH solution followed by calcination at 800 °C. Moreover, Figure 1C,D demonstrate SEM images of another sample (1.0 M KOH at 170 °C for 3 h and calcined at 600 °C). These images show that the product resembles multilayer graphene, as thin carbon layers are seen.

**Figure 2:**
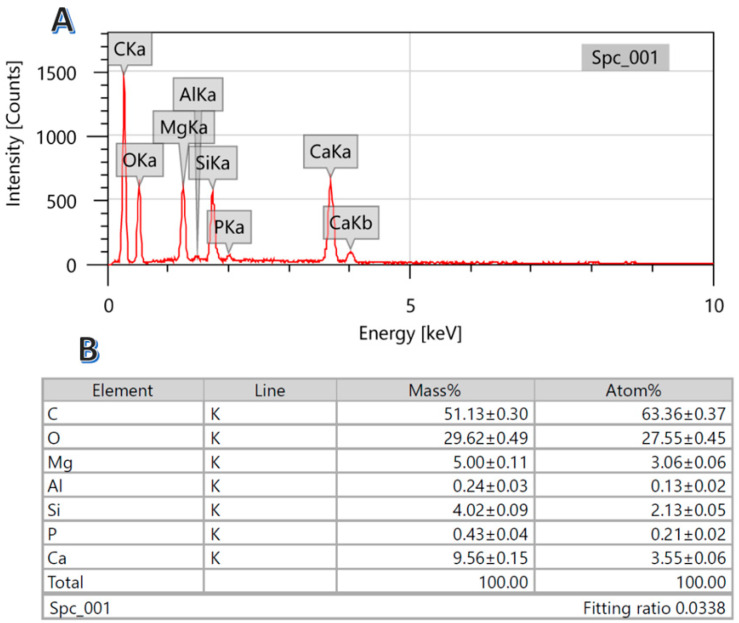
EDS spectrum; (**A**) and elemental analysis; (**B**) for a powder produced from hydrothermal treatment using 3.0 M KOH at 150 °C for 6 h and calcined at 800 °C.

Energy-dispersive X-ray spectroscopy (EDS) analysis was invoked in order to check the elemental content in the hydrothermally treated corn stalk pith at 150 °C for 3 h using 3.0 M KOH solution calcined at 800 °C. As is shown in the obtained spectrum and the associated table (Figure 2), the results support the previous reports concluding that the corn stalk contains considerable amounts of silicon, magnesium, and calcium. Moreover, due to the removal of lignin during the alkali hydrothermal treatment process and the subsequent calcination process (which removed the volatile hydrocarbons), the carbon content in the final composite is not as high as in the original raw material. Also, some other elements could be detected in small amounts.

**Figure 3:**
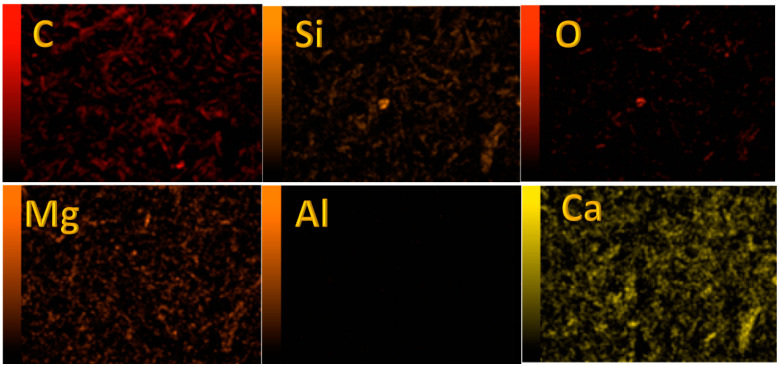
Elemental mapping for the produced powder after hydrothermal treatment in the presence of 1.0 M KOH at 170 °C for 3 h and calcined at 600 °C.

Figure 3 displays the elemental mapping of the proposed catalyst using 1.0 M KOH solution for 3 h at 170 °C). After careful washing and drying, the sample was sintered at 600 °C. As can be concluded from the color intensity in the obtained panels, besides carbon, calcium, magnesium, and silicon have high contents in the investigated sample. However, the sample has a negligible content of aluminum. These results are consistent with EDS analysis results; Figure 2. Noticeably, the oxygen distribution is very close to silicon, indicating that these two elements are bound. Furthermore, the calcium and magnesium distributions are also relatives, which reveals that these elements might be incorporated into a single chemical compound.

**Figure 4:**
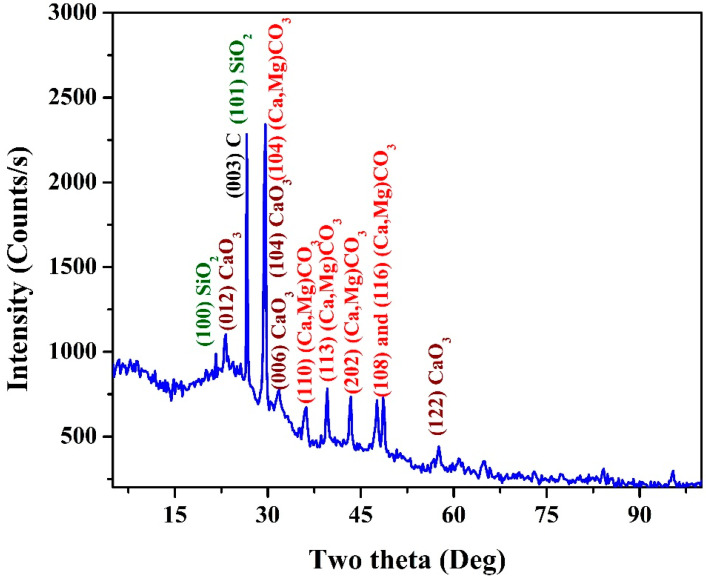
XRD pattern for a powder produced from hydrothermal treatment using 1.0 M KOH at 170 °C for 3 h and calcined at 600 °C.

X-Ray diffraction analysis (XRD) is a nondestructive technique which provides valuable and detailed information about crystalline materials’ chemical composition, crystallographic structure, and physical properties. It is based on the constructive interference of crystalline samples and monochromatic X-rays. In X-ray diffractogram analysis (XRD), the produced X-rays are collimated and directed at a sample. The interaction of the incident rays with the sample results in a diffracted beam, which is then detected, processed, and tallied. Therefore, it is a highly trustable analysis technique for checking the composition of crystalline materials (e.g., inorganic compounds). Figure 4 displays the XRD pattern for a sample obtained from a stalk pith. The observed peaks were assigned based on the EDS and elemental mapping results (Figure 2 and Figure 3). The peaks appeared at two theta values of 23.32°, 29.72°, 36.32°, 39.83°, 43.72°, 48.34°, and 49.15° corresponding to (012), (104), (110), (113), (202), (018), and (116) crystal planes, respectively, conclude the formation of a magnesium-rich calcite compound [(Ca, Mg)CO_3_] according to The International Centre for Diffraction Data (ICDD) card number 43-0697. This conclusion is supported by the similar distribution of Ca and Mg elements in the elemental analysis results. Moreover, pure calcite has been also detected, with some interfering peaks with (Ca, Mg)CO_3_. However, other peaks could be assigned, indicating the formation of pure calcite (ICDD 47-1743) as indexed in Figure 4. Additionally, based on ICDD (#46-1045), silicon metal has been compounded in the form of quartz, as it can be concluded from the appearance of the SiO_2_ standard peaks at two theta values 20.87°, 26.64°, 36.54°, 39.51°, 50.14°, 59.95°, and 68.15° corresponding to (100), (101), (110), (102), (112), (211), and (203) crystal planes, respectively. Similarly, the elemental mapping analysis supported the formation of silicon oxide, as the obtained images revealed similar distribution for the SiO_2_ formation elements; silicon and oxygen. Furthermore, the analysis proved the presence of carbon from the relatively broad diffraction peak which appeared at two theta values of 26.61° associated with the (003) crystal plane ICDD #26-1079. Due to the interference between the prominent diffraction peaks of the detected compounds, it is difficult to determine their mass or atomic percentages. However, it is safe to claim that the inorganic counterparts exist in high contents.

In XRD analysis, the prominent peaks representing graphite and graphene are close. A sharp peak assigns the graphitic structure at 2θ of 26.5° ((002) crystal plan) [35]. However, graphene is identified by a broadened peak at 2θ of 27.5° [36,37], indicating the small crystalline size of graphene in single or few layers. On the other hand, the presence of graphene oxide is accompanied by a strong diffraction peak at 2θ of 10.5° along with the disappearance of the graphite peak [35]. Therefore, it is difficult to identify the structure of the detected carbon in the prepared sample from the obtained XRD pattern; Figure 4. Raman microscopy compares graphite, graphene oxide, and graphene. In the case of graphite, a prominent peak (G band) appears at around 1580 cm^−1^, which corresponds to the first-order scattering of the E2g mode [38]. On the other hand, in the representative Raman spectrum of graphene oxide, the G band becomes broader and shifts to 1594 cm^−1^.

**Figure 5:**
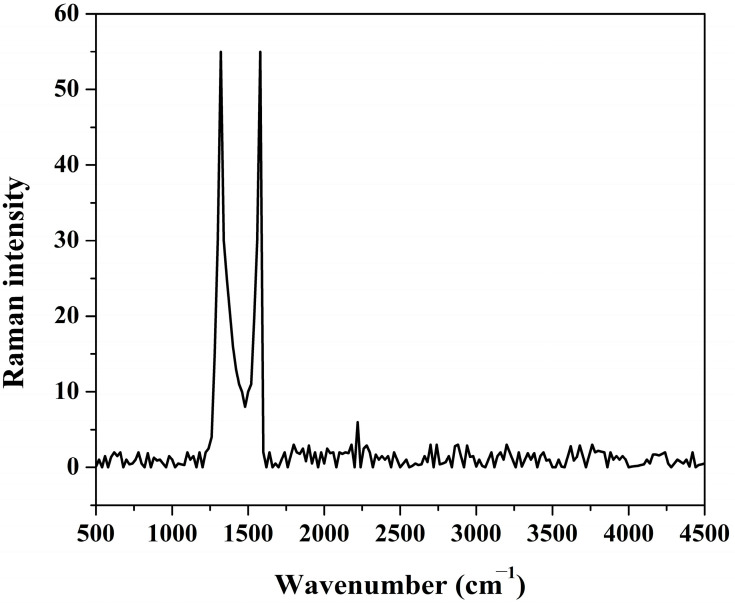
Raman spectroscopy spectrum for the prepared powder produced from hydrothermal treatment using 1.0 M KOH at 170 °C for 3 h and calcined at 600 °C.

Furthermore, in the graphene oxide Raman spectrum, because of the reduction in the size of the in-plane sp2 domains due to the extensive oxidation, another peak (D band) appears at 1363 cm^−1^ with high intensity. In the case of graphene, the corresponding Raman spectrum shows both G and D bands (at ~1580 and ~1350 cm^−1^, respectively) with an increase in the D/G intensities ratio compared to graphene oxide. The D/G intensities ratio is inversely proportional to the number of layers of the graphene sheets; it is very high with the monolayer graphene. This spectrum change was due to the decrease in the average size of the sp2 domains of graphene compared to graphene oxide [38]. Figure 5 demonstrates the Raman spectroscopy analysis of the prepared photocatalyst. As shown in the figure, two high-intensity peaks appeared at a wavenumber of 1324.8 and 1582.5 cm^−1^ which can be assigned to the D and G bands, respectively. This finding indicates that the carbon embedded in the investigated sample is composed of multilayer graphene sheets. In detail, the appearance of a high-intensity D band along with the G band (D/G ratio ~ 1) indicates the formation of graphene. However, detecting the G-band peak close to that of the graphite representative one concludes the construction of multilayer graphene [39].

**Figure 6:**
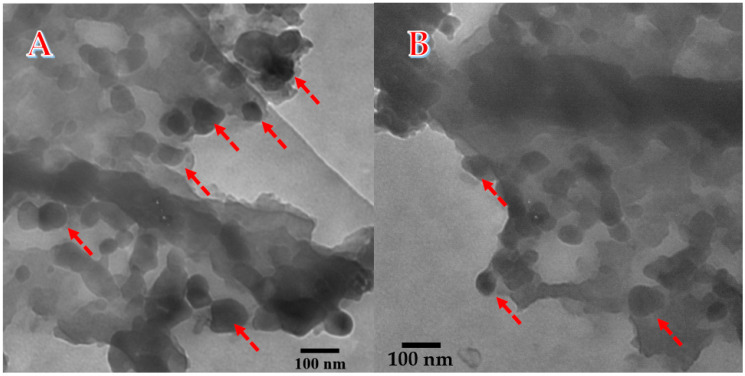
TEM image for a powder produced from hydrothermal treatment using 3.0 M KOH at 150 °C for 6 h and calcined at 800 °C; (**A**) and 1.0 M KOH at 170 °C for 3 h and calcined at 600 °C; (**B**). The arrows point to the crystalline inorganic NPs.

A transmission electron microscope (TEM) was utilized in order to analyze the detected inorganic compounds’ internal structure and distribution throughout the obtained graphene. Figure 6A demonstrates the obtained standard TEM image for a composite produced from the hydrothermal treatment of corn stalk pith at preparation conditions of 3.0 M KOH at 150 °C for 6 h and calcined at 800 °C. Moreover, Figure 6B displays the TEM image of the sample prepared under different conditions, with 1.0 M KOH at 170 °C for 3 h and calcined at 600 °C. As can be seen, for both formulations, the composite is in the form of multilayer graphene decorated by crystalline nanoparticles. Accordingly, from the utilized physicochemical characterization techniques, especially XRD and EDS, it can be claimed that the final product is in the form (Ca, Mg)CO_3_&SiO_2_ NPs-decorated multilayer graphene sheets. Typically, the deep black particles (pointed by red arrows) can be assigned to the crystalline inorganic nanoparticles attaching the graphene sheets, which is consistent with the literature [40,41].

**Figure 7:**
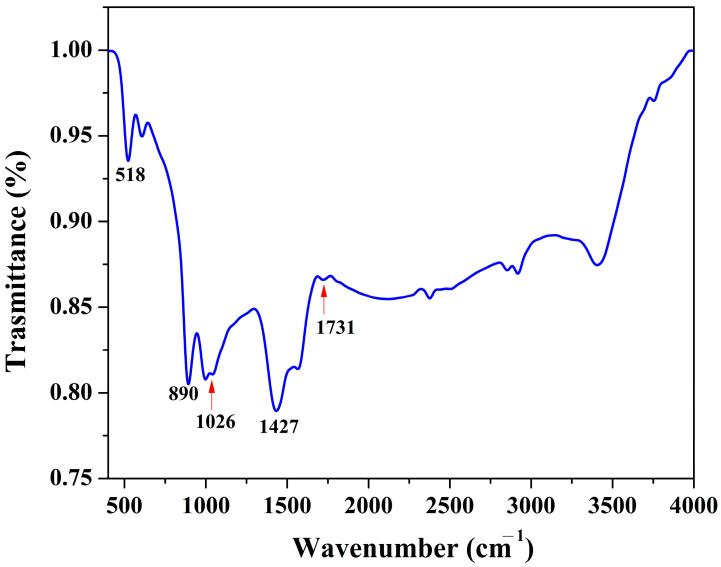
FTIR spectrum for a powder produced from hydrothermal treatment using 1.0 M KOH at 170 °C for 3 h and calcined at 600 °C.

Fourier transform infrared (FTIR) spectroscopy is a particularly useful analytical technique because it relies on objective criteria such as changes in the IR absorption frequency and intensity in various functional groups. Overall, there are five active IR lattice vibrations for calcite-type carbonates. Those are three vibrations relative to the center of gravity represented by the anti-translatory vibrations of class *E_u_* (when all the CO_3_ ions are vibrating in parallel or perpendicular opposite directions to the phase of the cations) and two liberations of the carbonate anion of class *A_2u_* [42]. These vibration peaks are usually observed at wavenumbers close to the observed peak in the obtained FTIR spectrum for the prepared sample (Figure 7) at ~518 cm^−1^. Other peaks are seen in the spectrum at 890, 1026, 1427, and 1731 cm^−1,^ which can be assigned to the following function groups: C-H (cellulose and hemicellulose), C-O stretching and C-O deformation, OH bending and C=O stretching. Therefore, high adsorption capacity is expected to form the prepared graphene support.

**Figure 8:**
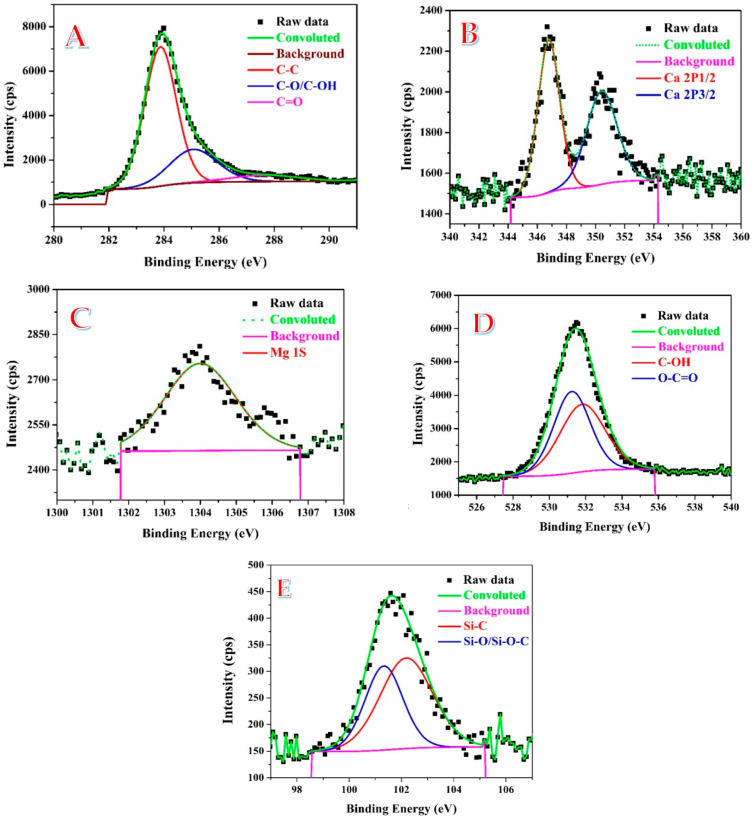
X-ray photoelectron emission spectroscopy of the C1s: (**A**), Ca2P; (**B**), Mg1S; (**C**), O1s; (**D**) and Si 2p; (**E**) core levels spectra for a powder produced from hydrothermal treatment using 1.0 M KOH at 170 °C for 3 h and calcined at 600 °C.

Using X-ray photoelectron spectroscopy, several elements’ chemical states and functional groups’ existences were further assessed (XPS). The presence of carbon was clearly visible in the survey scan spectrum of the investigated sample (prepared using 1.0 M KOH at 170 °C for 3 h and calcined at 600 °C). Moreover, in the survey, small peaks representing calcium, magnesium, and oxygen were also observed (the full survey spectrum is not shown). The C1s peak observed in the survey of the investigated powder was de-convoluted into three chemically shifted segments (Figure 8A) [43,44,45]. The first segment was attributed to C–C/C–H non-oxygenated carbon in the graphene structure [44,46]. The aforementioned TEM and XRD analyses could also support this conclusion. On the other hand, oxygen in either hydroxyl (C–OH) or epoxide (C–O) functional groups was assigned in the second segment representing the interaction of carbon atoms. However, the carbonyl (C=O) could also be identified in the third segment, which relates to either carbon functionalized in COOH or the carbonate group. Figure 8B displays the broad scan spectra of the Ca core-level spectra of the investigated powder. The two peaks observed in the Ca 2p region with binding energies of 346.8 and 350.5 eV are in perfect agreement with the reported characteristic peaks of Ca 2p1/2 and Ca 2p2/3, respectively [47]. The presence of magnesium was nicely detected by observing a single peak matching the convoluted data of the Mg 1S peak at a binding energy of 1304 eV, as shown in Figure 8C. Formation of the carbonate group could be also supported by the XPS spectra at the O 1S region. As shown in Figure 8D, a segment assigned to the O-C=O function group could be tasked with high intensity peak at a binding energy of 531.3 eV which concludes presence of the inorganic group [48]. Additionally, a phenolic (C-OH) group could be also detected within the O 1S region. Figure 8E displays the Si 2p line in the sample’s normalized XPS spectra. The Si 2p spectrum may be deconvolved into two primary components, corresponding to a Si-C bond at 101.2 eV and a Si-O/Si-O-C bond (silicon oxide/silicon oxycarbide) at 102.7 eV [49]. Therefore, it can be concluded that the detected SiO_2_ NPs are anchored with the graphene sheet by a Si-C bond [50].

A precise estimation of the band gap energy is necessary in order to forecast semiconductors’ photochemical and photophysical properties. When addressing semiconductor photocatalytic properties, band gap energy is widely utilized. Tauc suggested estimating the band gap energy of semiconductors using optical absorption spectra [51]. Davis and Mott explored his concept further [52]. The energy-dependent absorption coefficient (α) can be represented using the Tauc technique according to the equation below [53].

(1) (α·hv)1γ=B(hv−Eg)

*B* is a constant in this equation, while the letters *h*, *v*, and *E_g_* stand in for the Planck constant, the photon’s frequency, and the band gap energy, respectively. The *γ* factor, related to the kind of electron transition, has values of 0.5 and 2, respectively, for the direct and indirect transition band gaps. Typically, diffuse reflectance spectra are used to measure the band gap energy. However, the following procedure may be used to convert the reflectance spectra to the appropriate absorption spectra using the Kubelka–Munk function:

(2) $$F\left(R_{\infty }\right)=\frac{K}{S}=\frac{{\left(1-R_{\infty }\right)}^{2}}{2R_{\infty }}$$

where *K* and *S* are the absorption and scattering coefficients, respectively, and $R_{\infty }=R_{sample}/R_{standard}$ is the reflectance of an infinitely thick material. Updating Equation (1) by replacing *α* with *F*(*R*_∞_) results in this new equation form:

(3) (F(R∞)·hv)1γ=B(hv−Eg)

**Figure 9:**
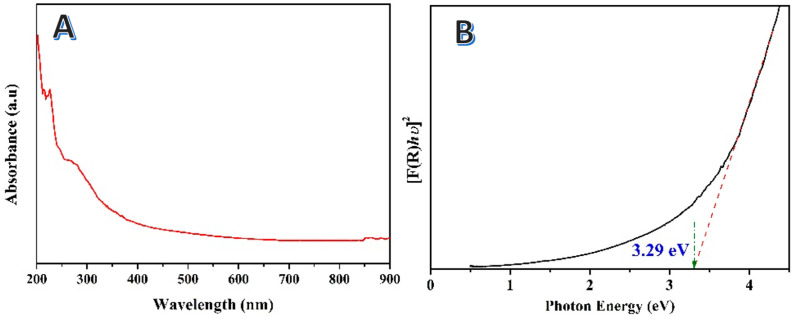
UV-vis spectra for the prepared photocatalyst from corn stalk pith hydrothermally treated by 1.0 M KOH at 170 °C for 3 h and calcined at 600 °C; (**A**), and Tauc plots for determination the band gap energies for the prepared sample; (**B**).

Figure 9A displays the UV-vis spectra for the prepared (Ca, Mg)CO_3_&SiO_2_ NPs-decorated multilayer graphene from corn stalk pith treated by 1.0 M KOH solution at 170 °C for 3 h and calcined at 600 °C. Figure 9B represents the reflectance spectra transformed according to Equation (3) and plotted against the photon energy for the prepared nanocomposite. When the incident light energy is increased, a section of the semiconductor exhibits a steep slope which indicates a linear rise in light absorption. The band gap energy can be estimated from the intersection point of the linear fit of the Tauc plot with the *x*-axis. As could be concluded from the figures, the prepared composite reveals higher band gap energy compared to the R25 titanium oxide nanoparticles. Typically, the band energies for the commercial TiO_2_ nanoparticles and proposed nanocomposite are 3.1 [54] and 3.29 eV, respectively. It is noteworthy that the obtained band gap is distressed if the prepared material composes a single-phase compound, because it suggests applicability as a photocatalyst under UV light irradiation. Fortunately, the prepared material is a nanocomposite. There are multiple individual band gaps; among them, it might be that one (or more) is consistent with the visible light radiation.

### 3.2. Hydrogen Production

#### 3.2.1. Effect of Preparation Parameters

To be an efficient photocatalyst, the proposed material should possess a conduction band potential level more negative than the redox potential of the lowest molecular orbital of the photocatalyst acceptor. On the other hand, the potential level of the valence band of the proposed photocatalyst should be more positive than the highest occupied molecular orbital reduction potential in order to start the oxidation process. This mainly controls the formation of hydrogen and oxygen on the photocatalyst surface. In other words, the photocatalyst’s valence and conduction band potentials should be wider than the oxygen and hydrogen generation potential levels for the H^+^/H_2_ and O_2_/H_2_O reactions to begin H^+^/H_2_ (−0.41 V vs. normal hydrogen electrode [NHE] at pH 7), O_2_/H_2_O (+0.82 V vs. NHE at pH 7) [55].

**Figure 10:**
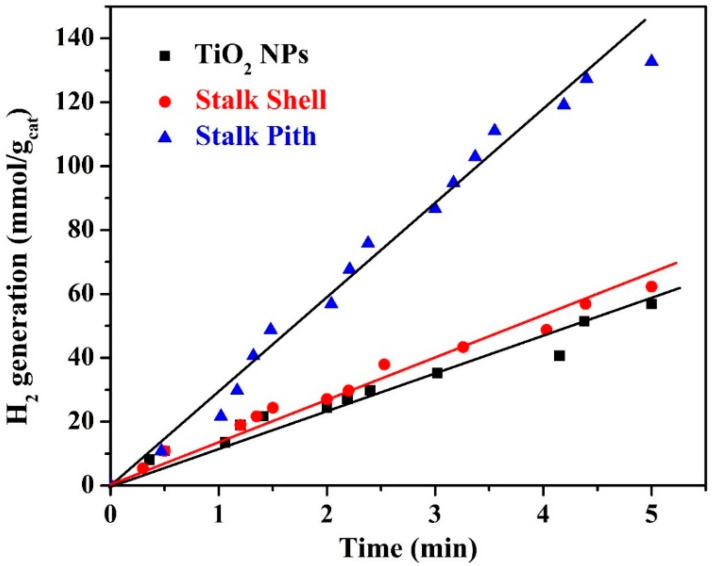
Hydrogen production rate from water splitting under visible light radiation using a catalyst produced from the shell and bulb of the corn stover. The photocatalytic performance is compared with that of TiO_2_ NPs (R25).

In reality, electron transmission is more complicated than what can be expected. Only at the electrolyte/semiconductor interface, where two orbitals belonging to the aqueous species and semiconductor possess equal energies, as has been demonstrated in previous studies, does electron transfer between semiconductors and aqueous redox species occur [56]. Accordingly, the composition and phase structure of the composite photocatalyst might have a distinct influence on the catalytic activity. Therefore, it is greatly expected that the initial composition of the biomaterial will considerably affect the photocatalytic activity of the prepared nanocomposite. For comparison, the proposed nanocomposite has been prepared from a stalk shell and stalk pith. In water photo-splitting reactions, titanium oxide can be considered a reference material to check the performance of the newly introduced photocatalysts. Figure 10 displays the hydrogen production rate using the prepared nanocomposite photocatalyst along with the results obtained using titanium oxide NPs. As shown in the figure, the nanocomposite prepared from the stalk pith has higher photocatalytic activity toward water splitting under visible light radiation than the standard photocatalyst and the nanocomposite produced from the stalk shell. Compared to cellulose and hemicellulose, which have a chain structure, lignin is a 3D molecule. Therefore, the latter usually exists in the complex parts of the plants to enhance their mechanical properties. Accordingly, it can be claimed that the lignin content in the stalk shell is higher and more complicated than that in the pith.

Consequently, the structure and composition of the two photocatalysts will be different, which will affect the photocatalytic activity accordingly. Compared to the used commercial titanium oxide nanoparticles, the stalk pith-obtained nanocomposite performs much better as a photocatalyst toward water photo-splitting, although it has higher band gap energy. Numerically, the hydrogen production rates were 11.95 and 28.78 mmol H_2_/gcat.min for nanocomposite photocatalysts obtained from the shell and pith of the corn stalk, respectively. At the same time, it was 10.27 mmol H_2_/g_cat_·min when titanium oxide NPs were used. This finding can be attributed to the multi-component composition of the nanocomposite, so some compounds act as primary photocatalysts and others as co-catalysts.

**Figure 11:**
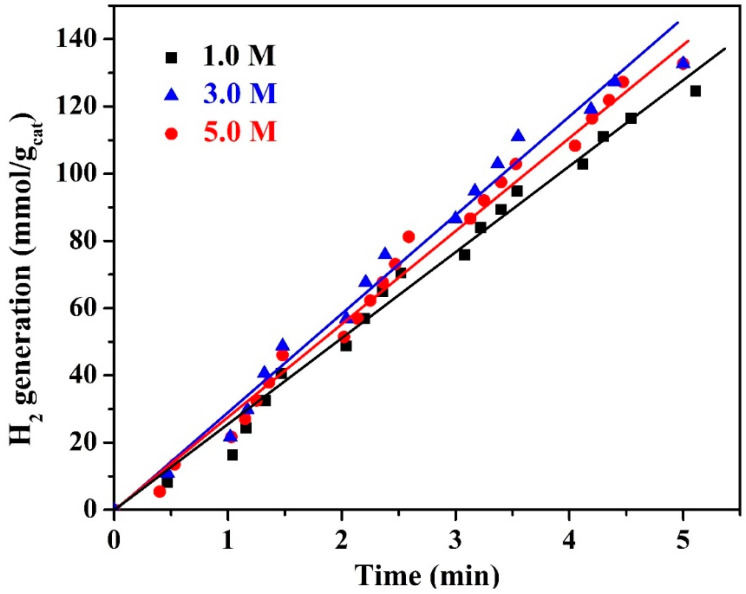
The influence of the KOH solution concentration on the hydrogen production rate from water splitting under visible light radiation; hydrothermal duration of 6 h, temperature of 150 °C, and calcination temperature of 800 °C.

The influence of the alkaline solution concentration on the corn stalk pith-prepared nanocomposite’s photocatalytic activity was investigated. Figure 11 displays the corresponding results. Increasing the potassium hydroxide concentration from 1.0 to 3.0 M slightly improved the hydrogen production rate. However, increased alkaline solution concentration negatively impacted the photocatalytic activity of the produced nanocomposite, as can be seen from the figure. Therefore, it can be concluded that at 1.0 M KOH concentration, hydrolysis of lignin and other aromatic compounds occurs at a proper level. However, the highest removal is carried out at 3.0 M concentration. A greater increase in the alkaline solution concentration leads to the breakdown of the cellulose and hemicellulose contents which negatively affects the final product performance. Mathematical calculations indicated that the hydrogen production rates are 26.09, 28.78, and 28.38 mmol H_2_/g_cat_·min for 1.0, 3.0, and 5.0 M alkaline solution concentrations, respectively.

**Figure 12:**
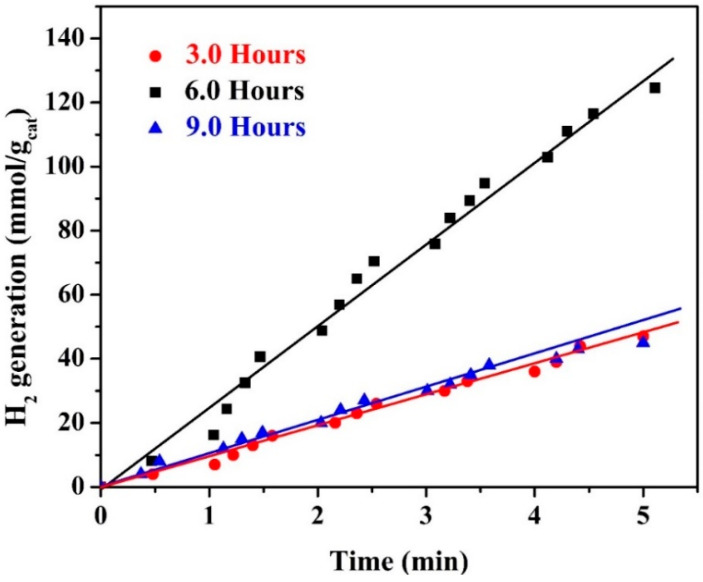
Influence of the hydrothermal treatment time on the hydrogen production rate from water splitting under visible light radiation; KOH concentration of 1.0 M, hydrothermal temperature of 150 °C, and calcination temperature of 800 °C.

In contrast to the alkaline solution concentration, studying the hydrothermal treatment time strongly influences the photocatalytic activity of the proposed nanocomposite, as shown in Figure 12. Numerically, at 6 h of hydrothermal treatment time, the estimated hydrogen production rate was 26.09 mmol H_2_/g_cat_·min. Meanwhile, for the two shoulders of the optimum hydrothermal treatment time (3.0 and 9.0 h), the observed hydrogen production rates were 9.67 and 9.25 mmol H_2_/g_cat_·min, respectively. The same trend of the alkaline solution concentration effect can explain these results. In detail, the best removal of the lignin and other undesired carbohydrates is achieved at 6.0 h of treatment time. At the same time, the long hydrothermal time (9.0 h) results in unrecommended cracking of the cellulose and hemicellulose chains. Breaking down the cellulose and hemicellulose chains affects the carbonaceous matrix’s morphology, functional groups, and structure in the obtained nanocomposite photocatalyst.

**Figure 13:**
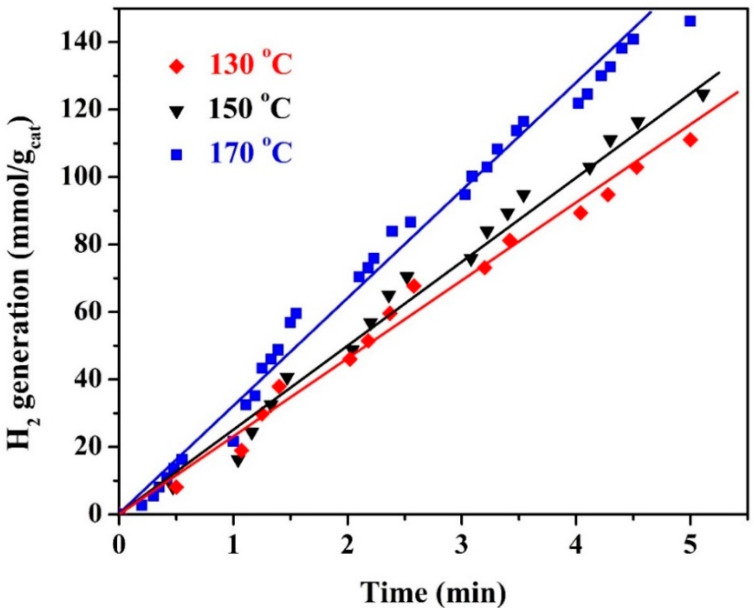
Influence of the hydrothermal treatment temperature on the hydrogen production rate from water splitting under visible light radiation; KOH concentration of 1.0 M, hydrothermal time of 3 h, and calcination temperature of 800 °C.

Increasing the hydrothermal treatment temperature is associated with increasing the reactor’s internal pressure. For instance, increasing the reaction temperature from 130 to 170 °C increases the reactor pressure from 2.66 to 7.83 atm, respectively. Therefore, for safety reasons, the experiments were conducted at a maximum temperature of 170 °C upon studying the hydrothermal reaction temperature effect. Typically, the influence of the reaction temperature was investigated by preparing the proposed nanocomposite at 130, 150, and 170 °C; the results are demonstrated in Figure 13. The obtained data indicate that the reaction kinetics of the suggested hydrothermal treatment process follow the general trend of normal chemical reactions. In other words, increasing the reaction temperature enhances the treatment reaction rates, which translates into improving the hydrogen production rates, as shown in Figure 13. As could be estimated from the figure, the hydrogen production rates are 22.53, 26.09, and 31.21 mmol H_2_/g_cat_·min, respectively.

**Figure 14:**
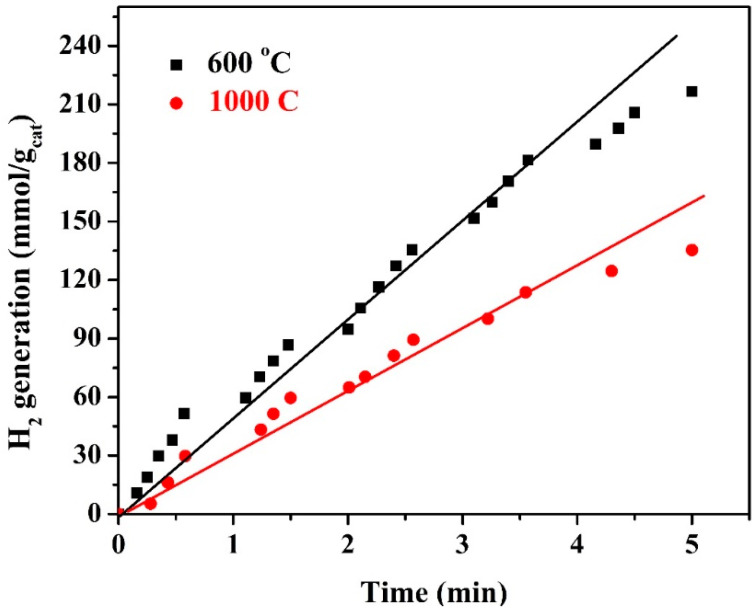
Influence of the calcination temperature on the hydrogen production rate from water splitting under visible light radiation; KOH concentration of 1.0 M, hydrothermal time of 3 h, and hydrothermal temperature of 170 °C.

Carbon supports for functional materials are usually used to exploit their adsorption capacity. In other words, for the heterogeneous catalytic reactions using carbon-based catalysts, the process is considered a combination between adsorption and the primary reaction [57]. Adsorption capacity mainly depends on functional groups located on the surface of the carbonaceous materials. As a result, functional group-free carbon materials usually have trivial adsorption capacity, and so the addition of various functional groups through proper activation processes is recommended in order to improve the adsorption capacity of the carbonaceous materials [58]. High-temperature thermal treatment process leads to losing the functional groups from the carbonaceous adsorbent supports, which negatively affects the adsorption capacity and decreases the activity of the whole composite consequently. The aforementioned hypothesis has been affirmed experimentally in Figure 14, which displays the effect of calcination temperature on the photocatalytic activity of the proposed catalyst. As shown in the figure, applying high calcination temperature shows inimical impact on the nanocomposite photocatalytic activity. This can be attributed to losing the functional groups from the graphene support; the observed hydrogen production rates are 43.35 and 27.89 mmol H_2_/g_cat_·min for the photocatalysts prepared at 600 and 1000 °C, respectively.

It is noteworthy that every experiment was conducted in three cycles, the introduced data are average values, and almost similar results were obtained in every cycle, which indicates the good stability of the proposed catalyst. This finding can be attributed to the expected stable structure of the prepared catalyst, as it is composed of stable compounds in the aqueous solution. Therefore, this finding also concludes the unchanged composition of the used catalyst. To properly evaluate the performance of the introduced photocatalyst, a comparison with some reported catalysts is presented in Table 2. As is shown, the introduced photocatalyst has priority.

#### 3.2.2. Photocatalytic Performance Measurement

The main metrics used to assess a particulate photocatalyst’s performance during overall water splitting are photocatalytic activity, quantum yield, and STH energy conversion efficiency. The quantity of gas that evolved during the illumination period or the average amount of gas produced per unit of illumination time can represent photocatalytic activity (the gas evolution rate). Specifying the light source strength, irradiation wavelength range, photocatalyst quantity, reactor type, reaction solution volume, and temperature is vital, since both rely heavily on experimental conditions. As the photocatalytic activity is not proportional to the mass of the photocatalyst under a specific set of reaction conditions, normalizing the evolution rate by the photocatalyst mass (e.g., mmol/g.min) is useless. Instead, the particular photocatalyst material and irradiation parameters control the connection between the photocatalytic rate and the amount of photocatalyst. An extensive debate on this subject has been published [71,72].

Quantum yield and solar-to-hydrogen (STH) energy conversion efficiency must thus be reported in order to evaluate photocatalyst performance and the current state of the art in the field of photocatalytic water splitting. The quantum yield is known as the ratio of photons contributing photocatalytic activity to all photons received. It is impossible to determine the precise quantity of photons absorbed by the photocatalyst because of light transmission and scattering. As a result, the apparent quantum yield (*AQY*), which can be determined using the equation below, is utilized.

(4) $$AQY=\frac{n\times R}{I}$$

where *n*, *R*, and *I* represent the number of electrons or holes utilized in forming a molecule of hydrogen or oxygen, the gas molecules’ quantity evolved in a specific time interval, and the incident photons number reached by the photocatalytic system during the stated time interval, respectively. Other researchers used STH to calculate the photocatalytic efficiency during water splitting process, which can be estimated by the following equation [71]:

(5) $$STH=\frac{R\times \Delta G}{P_{sun}\times S}$$

Here, *R*, Δ*G*, *P_sun_*_,_ and *S* denote the rate of hydrogen evolution during the water splitting reaction, the Gibbs energy of the water splitting reaction (273.13 kJ/mol), the energy flux of sunlight at the ASTM-G173 AM1.5 global tilt, and the irradiated photocatalyst area, respectively. The solar simulator is used not only in this study but also in many reports. In order to estimate the approximate STH values, the calculations were based on utilizing the energy flux of the utilized source of light (2000 watts); the results are summarized in Table 3 for all of the nanocomposites prepared in this study per 1 g of the used catalyst.

Finally, it is notable that hydrogen production from water splitting is still under debate [73]. The doubt about the effectiveness of water splitting operations is in respect to large-scale plants. However, the research is going on at a lab scale to introduce effective photocatalysts [74,75,76]. This study opens an avenue for a new class of photocatalysts extracted from agricultural wastes.

#### 3.2.3. Mechanism

From the literature, the band gap energies of CaCO_3_, MgCO_3_, and SiO_2_ are 5.07, 5.14, and 2.26 eV, respectively [77,78,79]. The conduction and valence bands of the prepared nanoparticles’ functional constituents must be identified in order to comprehend the water splitting mechanism employing the suggested catalyst fully. These empirical formulae might be used to predict the necessary bands:

(6) $$E_{CB}=ℵ-E^{e}-0.5E_{g}$$

(7) $$E_{VB}=E_{CB}+E_{g}$$

*E^e^* is the energy of the free electrons compared to hydrogen (about 4.5 eV), where *E_CB_* and *E_VB_* are the conduction and valance potentials, respectively (ca. 4.5 eV) [80]. The following equation can be used to measure the semiconductor’s absolute electronegativity (ℵ):

(8) ℵ=[x(A)a x (B)b x (C)c]1(a+b+c)

**Figure 15:**
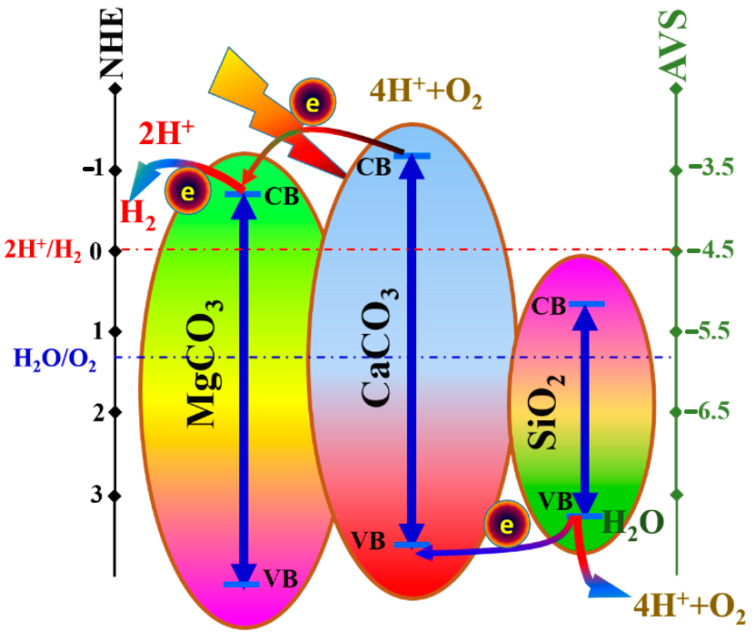
Schematic diagram showing the conduction and valance band locations in the prepared catalyst as well as illustrating the water splitting mechanism.

In this equation, *x*(*A*), *x*(*B*), and *x*(*C*) represent the absolute electronegativities of the elements *A*, *B*, and *C* in the compound *A_a_B_b_C_c_*, respectively. Accordingly, the absolute electronegativities of CaCO_3_, MgCO_3_, and SiO_2_ were calculated as 5.68, 6.32, and 6.47 eV, respectively. Therefore, the conduction and valance band pairs of these compounds could be determined from Equations (6) and (7) to be (−1.32, 3.68), (−0.75, 4.39), and (0.84, 3.1) eV in the same order. Figure 15 displays a conceptual illustration of the locations of the conduction valance bands in the prepared catalyst. From this diagram, the mechanism can be suggested. Typically, visible light is absorbed by CaCO_3,_ which was proven in the literature [81]. The existence of MgCO_3_ and SiO_2_ distinctly annihilates the electron/holes recombination process as the excited electrons will jump to the CB of MgCO_3_. In contrast, the holes will be more stable at the VB of SiO_2_, as is represented in the figure. Accordingly, hydrogen ions can be reduced at the CB of MgCO_3_, and H_2_O will be oxidized at the VB of SiO_2_ to produce hydrogen and oxygen, respectively.

## 4. Conclusions

X-ray diffraction (XRD), scanning electron microscope (SEM), transmission electron microscope (TEM), and X-ray photoelectron microscopy (XPS) analyses confirmed that the alkaline solution hydrothermal treatment process followed by calcination for corn stalk resulted in the formation of (Mg, Ca)CO_3_&SiO_2_ NPs-decorated graphene. However, as a photocatalyst for water splitting reactions, the experimental results indicated that utilizing the corn stalk pith precursor results in high performance compared to the stalk shell. Moreover, the results concluded that the alkaline solution concentration shows a relatively small impact on the photocatalytic activity of the produced nanocomposite. On the other hand, the hydrothermal time and temperature and the calcination temperature should be carefully adjusted in order to achieve high photocatalytic activity due to their considerable influence on the hydrogen production rate. Overall, this study opens a new avenue for the mineral-containing compounds existing in the biomasses to be exploited in the production of valuable and effective catalysts for hot applications.

## Figures and Tables

**Table 1:** Metal contents in the corn stover.

Element	Na	Mg	S	Si	Ni
Conc. (ppm)	6434 ± 27	5175 ± 50	2567 ± 8	36,239 ± 230	48 ± 0
Element	Ca	Al	P	Fe	K
Conc. (ppm)	11,670 ± 120	7333 ± 99	2761 ± 21	3808 ± 27	23,097 ± 61

**Table 2:** Hydrogen evolution rate for reported nanocatalysts.

Photocatalyst	Scavenger Agent	H_2_ (mmol/g_cat_·min)	Ref.
Pt/TiO_2_ nano sheet	Ethanol	0.0056	[59]
TiO_2_ NPs-Graphene	Methanol	0.0123	[60]
TiO_2_ nanoparticles	Methanol	0.1	[61]
Pt/HS-TiO_2_	Methanol	0.017	[62]
Pt-doped TiO_2_–ZnO	Methanol	0.0034	[63]
Pt-TiO_2_ particles	Methanol	0.444	[54]
Cd-TiO_2_ nanotube	Methanol	24	[64]
CdS/TiO_2_ mesoporous core-shell	Na_2_S/Na_2_SO_3_	1.13	[65]
Ni/TiO_2_ nanotube	-	0.433	[66]
Ni/GO-TiO_2_ NPs	Methanol	3	[67]
Ag-TiO_2_ NFs	Na_2_S/Na_2_SO_3_	2	[68]
NiCo_2_S_4_/CdO@CC	-	0.00125	[69]
Cd-TiO_2_ NPs Cd-TiO_2_ nanofibers	Na_2_S/Na_2_SO_3_	0.7 16.5	[70]
(Ca, Mg)CO_3_-SiO_2_ NPs-graphene	Methanol	43.35	This study

**Table 3:** STH values for the prepared nanocomposites photocatalysts prepared under different conditions.

Preparation Conditions				H_2_ Flow Rate (mmol/s)	STH (%)
KOH Conc. (M)	Hydrothermal Temp. (°C)	Hydrothermal Time (h)	Calcination Temp. (°C)		
1.0	150	6.0	800	0.434907	5.939307
3.0	150	6.0	800	0.47973	6.551435
5.0	150	6.0	800	0.473004	6.459579
3.0	150	3.0	800	0.161274	2.202443
3.0	150	9.0	800	0.154199	2.105814
1.0	130	6.0	800	0.375479	5.127724
1.0	170	6.0	800	0.520235	7.104592
1.0	170	3.0	600	0.722428	9.86584
1.0	170	3.0	100	0.464874	6.348554

## Data Availability

The data presented in this study are available upon request from the corresponding author.
